# Group B Streptococcus GAPDH Is Released upon Cell Lysis, Associates with Bacterial Surface, and Induces Apoptosis in Murine Macrophages

**DOI:** 10.1371/journal.pone.0029963

**Published:** 2012-01-23

**Authors:** Liliana Oliveira, Pedro Madureira, Elva Bonifácio Andrade, Abdelouhab Bouaboud, Eric Morello, Paula Ferreira, Claire Poyart, Patrick Trieu-Cuot, Shaynoor Dramsi

**Affiliations:** 1 Universidade do Porto, ICBAS-Instituto de Ciências Biomédicas de Abel Salazar, Porto, Portugal; 2 IBMC-Instituto de Biologia Molecular e Celular, Porto, Portugal; 3 Institut Cochin, Université Paris Descartes, Sorbonne Paris Cité, Faculté de Médecine, Centre National de la Recherche Scientifique (UMR 8104), Paris, France; 4 Institut National de la Santé et de la Recherche Médicale, U1016, Paris, France; 5 Institut Pasteur, Unité de Biologie des Bactéries Pathogènes à Gram-positif, CNRS URA 2172, Paris, France; Ecole Polytechnique Federale de Lausanne, Switzerland

## Abstract

Glyceraldehyde 3-phosphate dehydrogenases (GAPDH) are cytoplasmic glycolytic enzymes that, despite lacking identifiable secretion signals, have been detected at the surface of several prokaryotic and eukaryotic organisms where they exhibit non-glycolytic functions including adhesion to host components. Group B Streptococcus (GBS) is a human commensal bacterium that has the capacity to cause life-threatening meningitis and septicemia in newborns. Electron microscopy and fluorescence-activated cell sorter (FACS) analysis demonstrated the surface localization of GAPDH in GBS. By addressing the question of GAPDH export to the cell surface of GBS strain NEM316 and isogenic mutant derivatives of our collection, we found that impaired GAPDH presence in the surface and supernatant of GBS was associated with a lower level of bacterial lysis. We also found that following GBS lysis, GAPDH can associate to the surface of many living bacteria. Finally, we provide evidence for a novel function of the secreted GAPDH as an inducer of apoptosis of murine macrophages.

## Introduction

Group B Streptococcus (GBS, also known as *Streptococcus agalactiae*) is a common colonizer of the gastro-intestinal and urogenital tracts of up to 40% of healthy individuals. However, under certain circumstances, it may turn into a life-threatening pathogen causing sepsis and meningitis in newborn infants [1]. Mortality due to neonatal GBS infection remains high (up to 10%) despite antibiotic treatment and 25–50% of surviving infants are left with permanent neurological sequelae [2]. GBS is also responsible for invasive infections in adults with underlying diseases and in the elderly [1].

Glyceraldehyde 3-phosphate dehydrogenases (GAPDHs) are essential cytoplasmic enzymes involved in the glycolytic pathway which, despite the lack of secretion signals, have been found localized at the surface of several bacteria, fungi, and even protozoans (reviewed in [3]). In this unexpected location, GAPDH exhibits various adhesive functions thereby facilitating colonization and invasion of host tissues (reviewed in [3]). Like GAPDH, several other cytosolic proteins have been associated with the bacterial surface where they play a different role as referred to their original function and thus have been called “moonlighting proteins” (for review see [4]). Examples of moonlighting proteins include α-enolase [5], glucose-6-phosphate isomerase [6], glutamine synthetase [7], ornithine carbamoyltransferase [6] and fibronectin-binding proteins (PavA of *Streptococcus pneumoniae* [8], FbpA of *Listeria monocytogenes* [9], Fbp54 of *Streptococcus pyogenes* [10]).

Surface-localized GAPDH was originally identified in the Gram-positive pathogen *S. pyogenes* and subsequently found in other streptococcal groups B, C, E, G, H, and L [11]. *S. pyogenes* GAPDH is an ADP-ribosylating enzyme [12] that binds a number of human proteins, including plasmin(ogen) [13], [14], lysozyme, myosin, actin, fibronectin [11] and uPAR/CD87 on pharyngeal cells [15]. GAPDH has also been reported on the surface of Gram-negative bacteria such as enterohemorrhagic and enteropathogenic *Escherichia coli* where it binds to human plasminogen and fibrinogen suggesting a role in pathogenesis [16]. It is generally assumed that the release of such cytoplasmic proteins is due to cell lysis, although the involvement of specific export processes has been suggested [17], [18]. Nevertheless, the mechanism by how these proteins are exported, secreted or become surface associated is still a matter of debate.

GAPDH was also identified as a surface exposed and enzymatically active protein in GBS [19]. We have demonstrated that GAPDH is detected in the culture supernatants of GBS and acts as a virulence–associated immunomodulatory protein that exerts stimulatory effects on B lymphocytes and induces an early IL-10 production that facilitates host colonization [20]. We have also reported that surface-localized GAPDH interacts with the human plasminogen system to increase the proteolytic activity of the bacterial surface [21]. These results highlight the contribution of the extracellular form of GAPDH to GBS virulence.

Here, we addressed the question of GAPDH export to the cell surface of GBS strain NEM316. Overall, our data suggest that GAPDH presence in the extracellular medium is due to bacterial lysis. We also found that GAPDH is a very “sticky” protein that, following lysis, can associate to the surface of many living bacteria. Evidence for a novel function for GBS GAPDH as an inducer of apoptosis of murine macrophages is also provided.

## Results

### Surface display of GAPDH is impaired in pilus mutants of GBS

**Figure 1:**
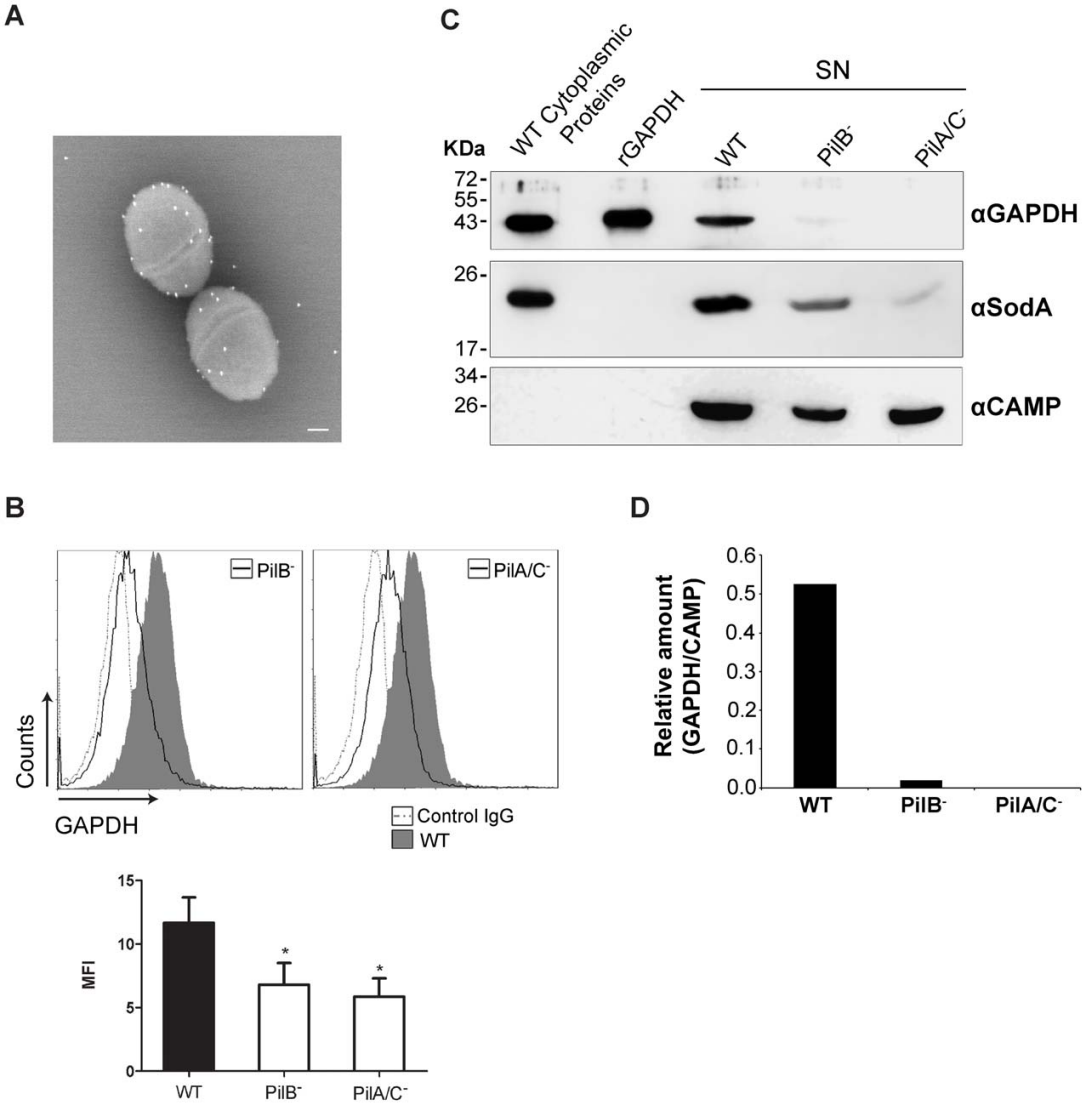
Evaluation of surface-bound and extracellular GAPDH levels in GBS strain NEM316 (WT) and pilus mutant strains. A) Immunoelectron microscopy (IEM) picture of the surface-bound GAPDH in NEM316. Scale bar, 100 nm. B) Fluorescence-activated cell sorter analysis (FACS) of WT, PilB^−^ and PilA/C^−^ incubated with a polyclonal rabbit anti-GAPDH IgG (grey filled histogram for WT or black line histogram for PilB^−^ and PilA/C^−^ strains) or normal rabbit IgG (control IgG-grey dotted histogram). Mean Fluorescence Intensity (MFI) in lower panel is reported relative to control IgG. C) Western Blot detection of GAPDH in culture supernatants (SN) of exponentially growing WT, PilB^−^ and PilA/C^−^ strains. 25 µL of each concentrated supernatant, 10 ng of rGAPDH or 2 µg of WT total proteins were loaded in the gel. After transfer to a membrane, proteins were detected using a polyclonal rabbit anti-rGAPDH IgG antibody, rabbit anti-SodA antibody and rabbit anti-CAMP antibody followed by HRP-conjugated goat anti-rabbit antibody. D) Quantifications of GAPDH relative to the loading control protein (CAMP) in the supernatant of WT, PilB^−^ and PilA/C^−^ strains, were performed with ImageJ software. Results shown are representative of three independent experiments. *, P<0.05.

We previously showed that GAPDH is present in the culture supernatant of GBS strain NEM316 [20] and immunogold electron microscopy revealed the presence of GAPDH on the surface of wild-type (WT) GBS strain NEM316 (Fig. 1A). However the mechanism of export of this abundant cytoplasmic protein devoid of signal sequence remains unknown. Screening our collection of *Himar1* transposon mutants [22] for the presence of surface displayed GAPDH, we found that the non-piliated PilB^−^ mutant [23], used as control, had an impairment in the presence of exposed GAPDH. Screening of additional mutants of the PI-2a pilus locus [23] unexpectedly revealed that mutants of the major pilin PilB^−^ and of both accessory proteins PilA/C^−^ exhibited a significant decrease in the amount of GAPDH present at the bacterial surface (Fig. 1B). In addition, we observed a significantly reduced level of GAPDH in the supernatants of these pilus mutants as compared to the WT strain (Fig. 1C). As an internal control for equal loading in western blots, we used antibodies specific for the CAMP factor, a known secreted GBS protein [24] and the GAPDH to CAMP ratio is shown in Fig. 1D. Of note, Western blot of these supernatant extracts with antibodies to the manganese-dependent superoxyde dismutase SodA, another GBS cytoplasmic protein [25], revealed that SodA protein is also found more abundantly in the culture supernatant of the WT strain compared to the pilus mutants (Fig. 1C). The presence of other cytoplasmic proteins was detected in the WT strain's culture supernatant using the available antibodies in the laboratory specific for known cytoplasmic proteins like D-alanine-D-alanyl carrier ligase (DltA) [26], NADH oxidase (Nox-2) [27], and elongation factor (EF-Tu) (Figure S1).

Some moonlighting proteins of *Mycobacterium tuberculosis* and *Listeria monocytogenes* were reported to be secreted *via* the accessory secretion machinery SecA2 [17], [18], [28] and we therefore tested the possibility that GAPDH export in GBS could be SecA2-dependent. FACS analysis and immunoblotting of culture supernatant proteins revealed that the GAPDH was slightly more abundant at the surface and in the culture supernatant of the SecA2 mutant as compared to the WT strain, thereby excluding the involvement of this secretion pathway in the export of this protein (data not shown).

### GBS pilus mutants display a lower level of bacterial lysis

**Figure 2:**
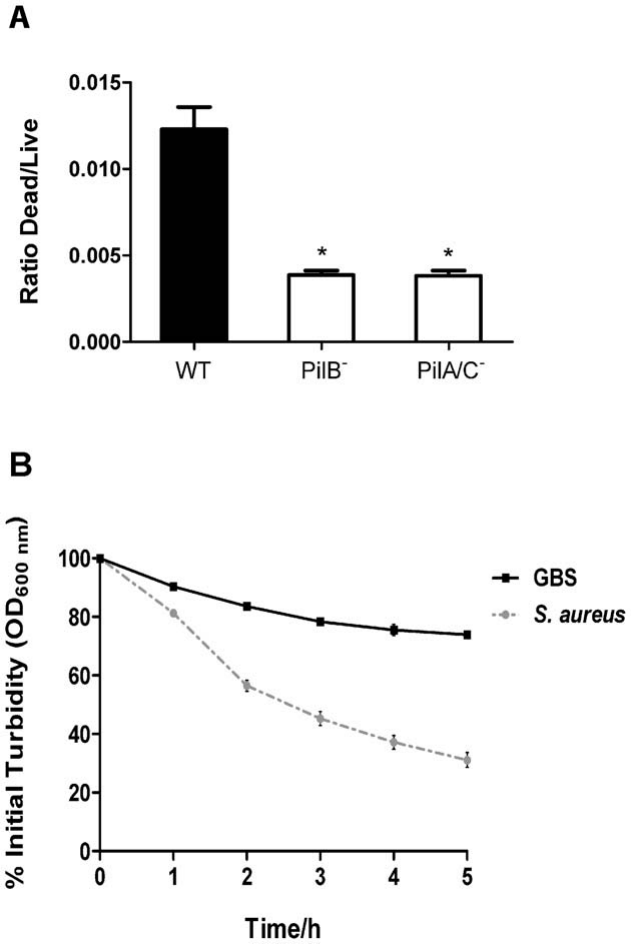
Quantification of bacterial lysis. A) 10 µL of bacterial culture of GBS WT, PilB^−^ and PilA/C^−^ were stained with Syto9 and propidium iodide (PI). The bacterial suspensions were then subjected to FACS and counted using the LIVE/DEAD® *Bac*Light™ kit. B) OD_600 nm_ measurement of GBS or *S. aureus* suspensions in PBS at 37°C over 5 h. The graphic expresses the % in OD in different time points relative to the time 0. Results shown are representative of two independent experiments. *, P<0.05.

We hypothesized that the secreted GAPDH both present on the surface and in the extracellular medium of the WT strain could result from bacterial lysis or to increased cellular permeability in membrane-compromised bacteria. We thus used the LIVE/DEAD bacterial viability kit for FACS to quantify bacterial lysis in WT and isogenic PilB^−^ and PilA/C^−^ pilus mutants. We found that the “Dead/Live” ratio was significantly higher in the WT strain compared to both pilus mutant strains (Fig. 2A). Thus, the WT strain appears more susceptible to lysis than PilB^−^ and PilA/C^−^ mutants. Of note, the *in vitro* growth curves of WT and pilus mutant strains were not significantly different (Figure S2). We compared the autolysis of GBS to that of *Staphylococcus aureus*, a bacterial species for which autolysis has been extensively studied including its impact on the release of cytoplasmic proteins [29]. In both species, autolysis was followed over time by measuring the decrease in turbidity of bacteria recovered in PBS (Fig. 2B). The graph shows a slow decrease in turbidity for GBS with a 20% decrease over 5 h. In contrast, a sharp decrease in turbidity was observed for *S. aureus*, dropping from 100 to ≈30% in the same time period. We estimated that the amount of GAPDH found in the supernatant of WT GBS represent approximately 5% of the amount of GAPDH present in total protein extracts (data not shown). Furthermore, in zymograms assays carried out with GBS, *Enterococcus faecalis* or *Micrococcus luteus* substrate gels, autolysin activity in GBS protein extracts was seen as a single faint hydrolysis band of about 60 kDa whereas *S. aureus* extracts gave 5–7 clear bands (data not shown). Thus, the low level of GBS autolysis, as demonstrated above, very likely accounts for the quantity of GAPDH found in the culture supernatant.

### Soluble GAPDH can associate to the bacterial surface of several Gram-positive and Gram-negative bacteria

**Figure 3:**
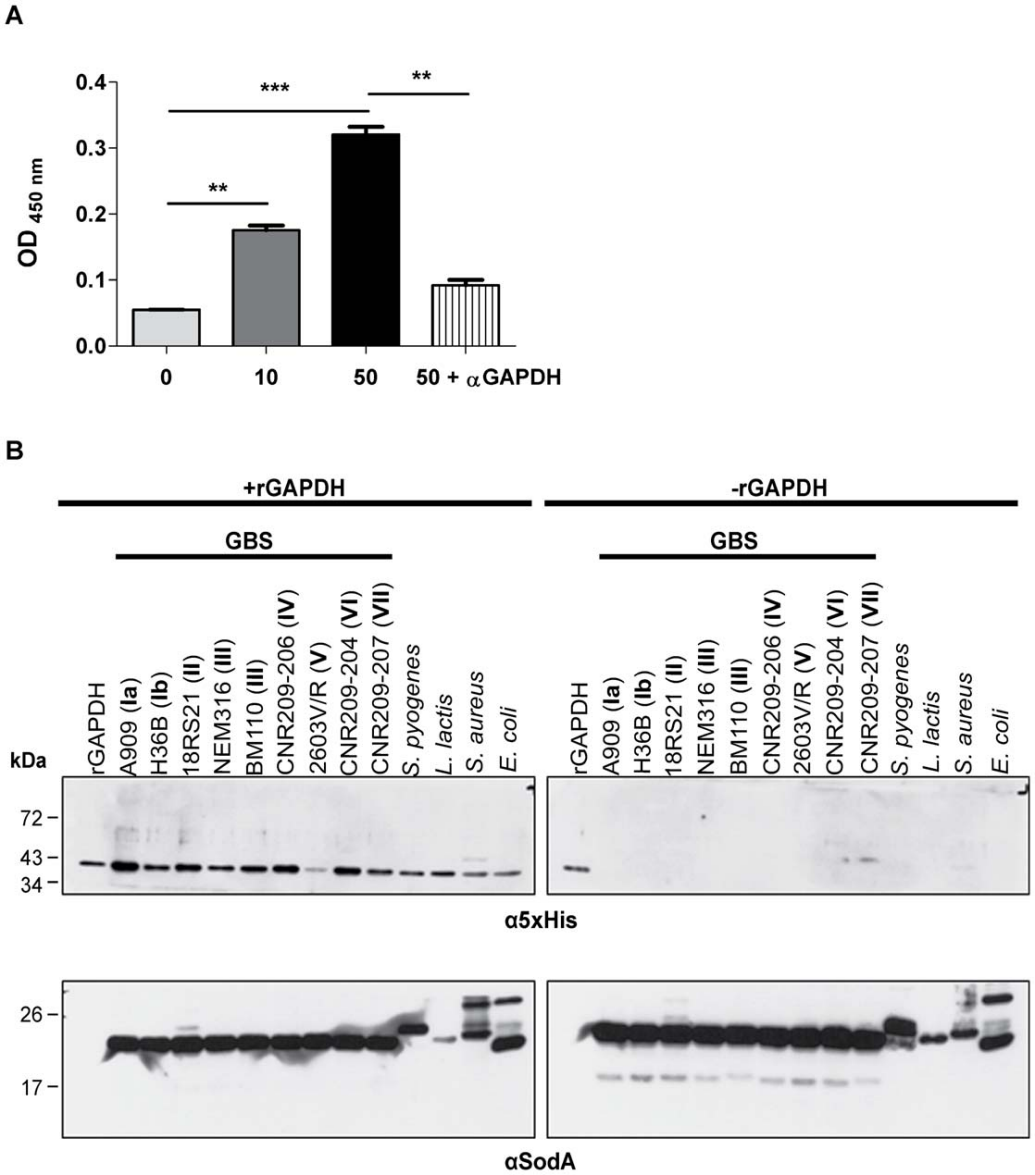
Reassociation capacity of rGAPDH. A) ELISA plates were coated with 6.5×10^7^ CFU/well of heat inactivated NEM316 overnight at 4°C. They were incubated with 100 µL/well of different amounts of rGAPDH (0, 10 or 50 µg/mL) or rGAPDH 50 µg/mL with 75 µg of rabbit anti-rGAPDH IgG pAb (50+αGAPDH). rGAPDH was detected using a HRP coupled mouse pentaHis antibody. After revelation, the OD_450_ was registered using a microplate reader. B) 200 µg of rGAPDH (+rGAPDH) or PBS (−rGAPDH) was added to an exponentially grown culture of several GBS strains belonging to different serotypes (Ia, Ib, II, III, IV, V, VI, and VII), *S. pyogenes*, *L. lactis*, *S. aureus*, and *E. coli*. After incubation, total proteins extracts were subjected to SDS-PAGE followed by transfer to nitrocellulose membrane (rGAPDH: 10 ng; Total proteins +/− rGAPDH: 8 µg). This membrane was incubated with an HRP-coupled mouse pentaHis antibody or rabbit anti-SodA antibody (loading control) followed by HRP-conjugated goat anti-rabbit secondary antibody. Results shown are representative of two independent experiments. **, P<0.01; ***, P<0.001.

The possibility that these housekeeping enzymes could become surface exposed by a process of reassociation was proposed previously [30]. In order to test the capacity of soluble GAPDH to bind to the bacterial surface, we performed an ELISA assay in which heat-inactivated GBS were used as a target to bind recombinant histidyl-tagged GAPDH (rGAPDH) added at different concentrations. The interaction between heat-inactivated GBS and rGAPDH was detected using a mouse anti-5×His antibody. The results indicated that rGAPDH interacts with the bacterial surface in a dose dependent manner and that this binding can be inhibited by anti-GAPDH antibodies (Fig. 3A). In addition, we performed another assay in which rGAPDH was added to exponentially growing bacteria including GBS strains from different serotypes, unrelated Gram-positive bacteria (*S. pyogenes*, *L. lactis*, and *S. aureus*), and *E. coli* as a Gram-negative prototype. Following 2 h of incubation at 37°C, bacteria were pelleted by brief centrifugation, washed three times with PBS, and protein extracts were analyzed by immunoblotting using a pentaHis antibody to detect the rGAPDH. This analysis showed that exogenous rGAPDH could efficiently bind to the surface of all the strains tested, including the phylogenetically remote Gram-negative bacterium, confirming the high propensity of this protein to interact with unrelated bacterial surface components (Fig. 3B).

### GAPDH can bind to the pilus structural subunits

**Figure 4:**
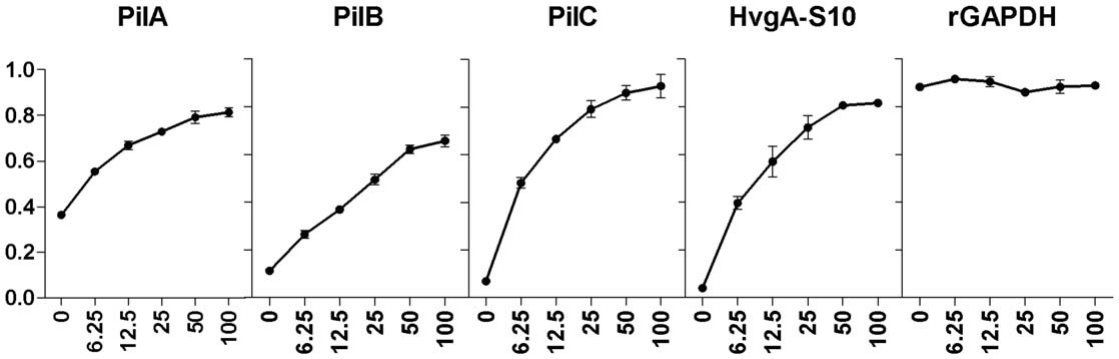
Interaction of rGAPDH with pilus structural subunits. ELISA plates were coated with 10 µg/mL of PilA, PilB, PilC, S10 domain of HvgA (HvgA-S10) or rGAPDH proteins. These plates were incubated with 100 µL/well of different amounts of rGAPDH (0, 6.25, 12.5, 25, 50 or 100 µg/mL). GAPDH was detected using a rabbit anti-rGAPDH IgG pAb. After the revelation, the OD_450_ was registered using a microplate reader. Results shown are representative of two independent experiments.

To test whether pilus subunits can act as GAPDH reassociation partner on the surface of GBS, we examined the direct interaction of GAPDH with purified histidyl-tagged pilus proteins, PilA-6×His, PilB-6×His, and PilC-6×His by ELISA. As control for specificity, we used another histidyl tagged protein His-HvgA S10 (S10 domain of HvgA protein [31]). Fig. 4 shows that although GAPDH was able to bind to all three pilus structural proteins, PilA, PilB and PilC, in a dose dependent manner, it similarly binds to the control protein S10-HvgA. These results indicate that GBS GAPDH is a very “sticky” protein that binds non-specifically to structurally unrelated surface proteins.

### Induction of bacterial lysis increases the amount of surface GAPDH

**Figure 5:**
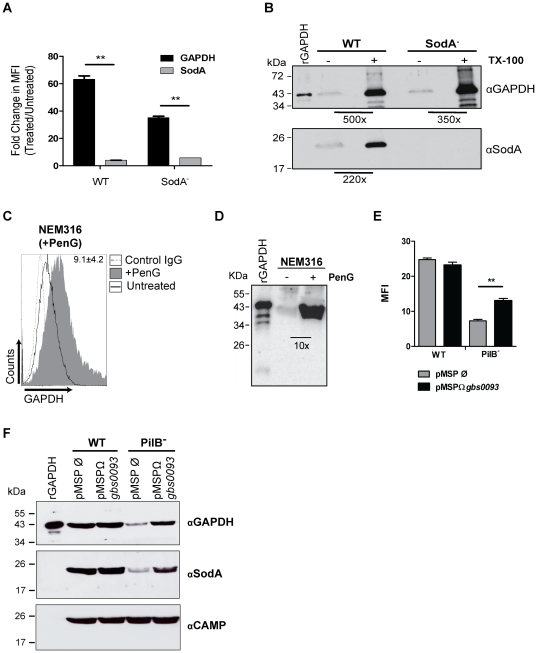
Release of cytoplasmic proteins upon induced bacterial lysis. A, B) NEM316 WT or SodA mutant cultures were treated (+) with 15 U/mL mutanolysin and 0.1% Triton X-100 or left untreated (−). The amount of surface GAPDH or SodA was determined by FACS with a rabbit IgG anti-rGAPDH pAb or anti-SodA followed by AlexaFluor 488-conjugated anti–rabbit antibody (A). The graphic expresses the fold change in MFI after treatment. B) Western Blot analysis of GAPDH and SodA proteins from NEM316 WT or SodA mutant detected using a rabbit anti-rGAPDH IgG pAb or rabbit anti-SodA followed by HRP-conjugated goat anti-rabbit antibody. rGAPDH: 10 ng; supernatants from untreated bacteria: 25 µL; supernatants from Triton-treated bacteria: 1 µL. Numbers in the western blot represent the fold increase of GAPDH or SodA amount after Triton treatment, as determined densitometrically and corrected for sample amount. C, D) 5×10^6^ CFU/mL of NEM316 were treated for 12 h with Penicillin G (+PenG) at 100× MIC (6.4 µg/mL) or left untreated. The bacteria were then subjected to FACS analysis as previously described (C). The numbers in the histogram represent the fold change in MFI after the treatment with PenG. D) Western blot analysis of GAPDH protein from NEM316 WT detected using a rabbit anti-GAPDH IgG pAb followed by HRP-conjugated goat anti-rabbit antibody. rGAPDH: 10 ng; supernatants: 25 µL. Numbers in the western blot represent the fold increase of GAPDH amount after PenG treatment, as determined densitometrically. E,F) GBS WT and PilB^−^ strains containing the inducible vector pMSP3545 empty (pMSP Ø) or encoding *gbs0093* (pMSPΩ*gbs0093*) were subjected to overnight induction with 20 ng/µL nisin after reaching an OD_600_ = 0.3. After this period, the amount of surface exposed GAPDH was quantified by FACS using anti-GAPDH antibodies (E) and 15 µL of supernatant extracts were analyzed for the presence of GAPDH, SodA, and CAMP by western blot (F). Results shown are representative of at least three independent experiments. **, P<0.01.

To substantiate the hypothesis that secretion of GAPDH results from cell lysis, we investigated whether induced GBS lysis resulted in increased amount of surface exposed GAPDH. For that purpose, GBS WT or the isogenic SodA mutant were incubated in the presence of 0.1% Triton X-100 for 45 min. As shown in Fig. 5A, Triton treatment increased by approximately 60-fold and 35-fold the amount of GAPDH detected by FACS analysis at the surface of the WT and SodA^−^ strains, respectively. Immunoblotting of supernatant proteins showed that Triton treatment increased by approximately 500-fold and 350-fold the amount of GAPDH detected in the culture medium of WT and SodA^−^ strains, respectively, as compared to the non-treated cultures (Fig. 5B). Interestingly, a 220-fold increase in the amount of SodA was also detected in the culture supernatant of the WT strain following Triton treatment. However, as shown in Fig. 5A, the moonlighting protein SodA does not reassociate to the bacterial surface.

We then reasoned that antibiotics targeting the cell wall, such as penicillins, and inducing cell lysis should also increase the release of GAPDH. Similar experiments were therefore performed with penicillin G (PenG), an antibiotic commonly used to treat GBS infections. Incubation for 12 h of exponentially growing GBS with PenG at a final concentration of 6.4 µg/mL (i.e. a therapeutic concentration corresponding to 100 times the MIC) resulted in a CFU/mL decrease of 5×10^6^ to 2×10^4^. Accordingly, after this treatment, there was approximately a 10-fold increase in the amount of GAPDH detected both at the cell surface (Fig. 5C) and in the culture supernatant (Fig. 5D).

To further confirm that GAPDH is secreted upon cell lysis we overexpressed one putative GBS autolysin, an N-acetyl muramidase, encoded by the gene *gbs0093*, in the WT and PilB^−^ mutant. Our first attempts in cloning *gbs0093* on high- and low-copy plasmid pOri23 and pTCV-*erm* respectively were unsuccessful. We therefore choose to assay an inducible system that has been used in closely related Gram-positive species using the pMSP3545 vector [32]. We first validated the functionality of this system in GBS NEM316 using the secreted staphylococcal nuclease NucB as a reporter. Addition of nisin in the culture medium allowed the detection of secreted NucB in the supernatant in a dose dependent manner without impairing bacterial growth (Figure S3).

Cloning of *gbs0093* in plasmid pMSP3545 was performed in *E. coli* and the resulting vector was introduced in WT and PilB^−^ GBS strains. We confirmed the overexpression of the autolysin after a 3 h induction with nisin by semi-quantitative RT-PCR with RNA extracted from both strains (data not shown). We next analyzed the amount of surface bound GAPDH by FACS and observed a significant increase of this protein in the PilB^−^ mutant after the overexpression of the autolysin following an overnight induction (Fig. 5E). In the WT strain, which display a higher level of bacterial lysis than the PilB^−^ mutant, the effect of autolysin overexpression was not statistically significant. We also verified by immunoblotting that GAPDH accumulated at higher levels in the culture supernatant of the PilB^−^ strain (Fig. 5F). Similarly, the amount of the SodA protein, used as control, also increased in the culture supernatant of the PilB^−^ strain when the autolysin was overexpressed.

### rGAPDH induces apoptosis in macrophages

**Figure 6:**
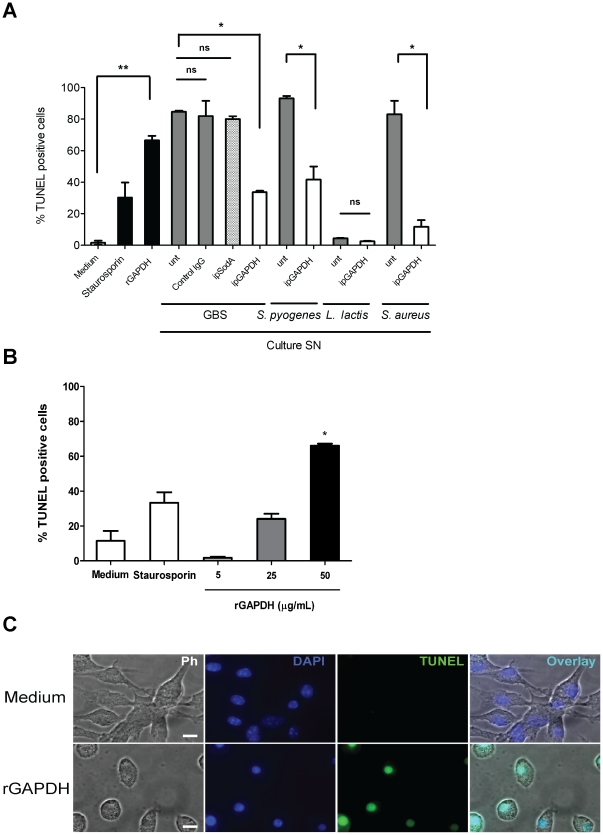
Induction of apoptosis mediated by (r)GAPDH. A) 5×10^5^ RAW264.7 murine macrophages were left untreated (Medium) or treated for 24 h with 1 µM staurosporin or 50 µg/mL rGAPDH, or 200 µL of culture supernatant from GBS, *S. pyogenes*, *L. lactis* or *S. aureus* left untreated (unt) or immunoprecipitated with rabbit anti-GAPDH IgG pAb (ipGAPDH), with rabbit anti-SodA IgG pAb (ipSodA) or with a normal rabbit IgG (control IgG). B) 5×10^5^ Bone marrow-derived macrophages from C57BL/6 mice were left untreated (Medium) or treated for 24 h with 1 µM staurosporin, or rGAPDH at the indicated concentrations. After treatment, the macrophages were fixed and stained for TUNEL using the Promega DeadEnd™ Fluorometric TUNEL kit. Samples were acquired and the % of cells that incorporated fluorescein-dUTP (TUNEL positive cells) was quantified. C) For IF, 1×10^5^ C57BL/6 bone marrow-derived macrophages were left untreated or treated for 24 h with 50 µg/mL rGAPDH. After that period, cells were fixed and stained as mentioned in B) and visualized in a fluorescence microscope. Scale bars, 15 µm. Results shown are representative of two independent experiments. ns, non-significant; *, P<0.05; **, P<0.01.

It was previously shown that GBS induces apoptosis of macrophages [33] and that eukaryotic GAPDH plays a role in the induction of cell death of different cell lines (reviewed in [34], [35]). Therefore, we investigated whether streptococcal GAPDH could induce apoptosis in both immortalized murine macrophages (RAW264.7) and primary bone marrow-derived macrophages from C57BL/6 mice. As shown in Fig. 6A, rGAPDH or culture supernatant from GBS WT strain induced efficient apoptosis in RAW264.7 at 24 h as determined by using a fluorometric TUNEL assay. As polymyxin B was added in the media containing recombinant proteins and bacterial culture supernatants to neutralize traces of LPS, we observed that its presence at the same concentration (10 µg/ml) in the medium only did not increase the levels of apoptotic cells (data not shown). In addition, immunodepletion of the GAPDH from the culture supernatant significantly decreased apoptosis level. This reduction in apoptosis level was not observed by immunodepleting the SodA protein (Fig. 6A). This result shows that native GAPDH found in the bacterial supernatant of GBS is a potent inducer of apoptosis in this murine cell line. Furthermore, we tested the capacity of the GAPDH present in the supernatant from both pathogenic (*S. pyogenes* and *S. aureus*) and non-pathogenic bacteria (*L. lactis*) to induce apoptosis of macrophages. Interestingly, the GAPDH of both *S. pyogenes* and *S. aureus* plays a significant role in macrophage apoptosis (Fig. 6A). In contrast, the culture supernatant from *L. lactis* was unable to cause apoptosis of macrophages. Similar results were obtained in primary bone marrow-derived macrophages where rGAPDH induced a marked pro-apoptotic effect which was shown to be dose-dependent (Fig. 6B). Moreover, the immunofluorescence images of C57BL/6 bone marrow-derived macrophages that were treated with rGAPDH (Fig. 6C) show the nuclear TUNEL staining and the classical morphological characteristics of apoptosis such as decreased size, a marked round shape and condensed nuclei.

## Discussion

Several reports in the last decade have shown that housekeeping enzymes, including glycolytic enzymes such as GAPDH, α-enolase (Eno), phosphoglycerate kinase (PGK), fructose 1,6-biphosphate aldolase (FBA), can mediate a variety of unrelated functions depending on their location in the cell and are therefore collectively referred to as “moonlighting proteins”. These cytoplasmic enzymes, which lack typical signature sequences for their transport to the cell surface and/or anchoring mechanisms to the cell wall have been observed at the surface of bacteria, yeast, fungi and protozoa (reviewed in [3]).

Seifert *et al*. first characterized GBS GAPDH as an anchorless surface adhesin conferring to the bacteria the ability to bind plasminogen and fibrinogen [19]. We next reported that GAPDH isolated from culture supernatant of GBS strain NEM316 has immunomodulatory properties, contributing to GBS evasion from the host immune system [20]. Interestingly, a GBS strain overexpressing GAPDH showed increased virulence as compared with the wild-type strain in C57BL/6 mice [20]. Furthermore, maternal vaccination with GAPDH conferred protection against GBS infection in neonatal mice [36].

NEM316 pilus, encoded by the PI-2a locus (i.e., *gbs1479-1474*), is composed of three structural subunit proteins: PilA (Gbs1478), PilB (Gbs1477), and PilC (Gbs1474) whose assembly involves two class C sortases (SrtC3 and SrtC4). PilB, the *bona fide* pilin, is the major component; PilA, the pilus associated adhesin, and PilC, are both accessory proteins incorporated into the pilus backbone. In this work, we unexpectedly found that the non-piliated PilB^−^ mutant derived from the WT strain NEM316 displays a decrease in surface GAPDH and decided to understand the molecular bases underpinning this observation.

### GAPDH presence on the cell surface is associated with bacterial lysis

Here we showed that the reduced amount of surface GAPDH, in both PilB^−^ and PilA/C^−^ strains, was due to a lower level of lysis compared to the parental strain NEM316 as determined by dead/live ratio. By comparing the amount of GAPDH found in the cytoplasm and supernatant, we estimated that ≈5% of bacterial lysis would account for the amount of extracellular GAPDH detected in the culture medium. This low level of autolysis is consistent with our turbidity assay showing a 20% decrease over 5 h (Fig. 2B). However, no significant difference in autolysis was observed between GBS WT strain NEM316 and the PilB^−^ and PilA/C^−^ mutants using the turbidity or zymogram assays which might simply reflect the poor sensitivity of these techniques. There is circumstantial evidence that some moonlighting proteins from *Mycobacterium tuberculosis* [17] and *L. monocytogenes* [18], [28] might be secreted *via* the SecA2 pathway. In staphylococci and streptococci including GBS, where it has been extensively studied, the SecA2 pathway is required for export of a serine-rich repeat protein, Srr1, containing a long atypical signal sequence [37], [38], [39], [40]. Interestingly, the secretion of two major autolysins with signal peptides (MurA and P60) identified in *L. monocytogenes* is SecA2-dependent [18], [41]. We therefore hypothesized that the release of moonlighting proteins (SodA, GAPDH, DNAK, GroEL, EF-Tu) in this bacterial species is a direct consequence of the cell lysis resulting from the activity of these SecA2-dependent autolysins, as recently demonstrated for the SecA-dependent autolysin Atl in *S. aureus* [29]. Indeed, Atl was recently reported to play a crucial role in the excretion of 22 cytoplasmic proteins, including GAPDH [29]. Our results rule out the possibility that the SecA2 machinery is involved in the export of GBS GAPDH (data not shown). Furthermore, we were able to detect several other classical cytoplasmic proteins like DltA, Nox-2 and EF-Tu in the supernatant of wild-type strain reinforcing our hypothesis of autolysis (Figure S1).

Interestingly, we found that addition of penicillin G significantly increased the amount of GAPDH in the culture supernatant (Fig. 5D) confirming that bacterial lysis contributes to GAPDH presence extracellularly. ß-lactam-induced toxin release is well-documented in streptococci and staphylococci and there is a recommendation to use these antibiotics in association with an inhibitor of protein synthesis, such as clindamycin, to suppress endotoxin release by bacteria [42]. Penicillin G, an antibiotic commonly used to treat GBS infections might therefore exert an antagonist effect to its bactericidal activity by favoring GAPDH display on the bacterial surface that in turn may favor invasive GBS colonization, an hypothesis that remains to be tested *in vivo*.

The autolytic process and its molecular bases have not been studied in GBS. *In silico* analysis of the NEM316 genome revealed the existence of five genes encoding putative autolysins: *gbs0092* (similar to D,D-carboxypeptidase), *gbs0093* (similar to N-acetyl muramidase), *gbs0678* (similar to beta-N-acetylglucosaminidase), *gbs1540* (similar to amidase or hydrolase), and *gbs1660* (similar to putative amidase). Our transcriptome data on strain NEM316 indicate that *gbs0093* is highly expressed which is confirmed by analysis of surface proteome (our unpublished data). We therefore decided to explore the role of this gene in autolysis using two approaches: construction of an in-frame deletion mutant of *gbs0093* in strain NEM316 and overexpression of this gene in the WT and PilB^−^ strains. The *Δgbs0093* mutant was not significantly different from the WT strain for surface display of GAPDH (data not shown) suggesting that some of these putative autolysins have redundant activities. In contrast, overexpression of *gbs0093* in the PilB^−^ mutant, a mutant displaying a lower level of bacterial lysis than the WT strain, led to an increased amount of GAPDH in the culture supernatant that perfectly correlated with a higher level of surface GAPDH in this strain (Fig. 5E, 5F). All together, these results indicate that GBS GAPDH is released following bacterial lysis and can then bind or remain attached to the surface of viable bacteria.

### GAPDH interacts with various proteins and many bacterial surfaces

GAPDH is a very “sticky” protein and its ubiquitous adhesive properties were characterized with a recombinant GAPDH that efficiently bind to pilus subunits, PilA, PilB and PilC and to other unrelated surface proteins, such as HvgA (Fig. 4). Of note, *S. pyogenes* GAPDH was shown to be associated with the M or M-like protein and this association may allow GAPDH to indirectly acquire plasmin generated by the M-protein/fibrinogen-mediated fibrinolytic complex [14]. We similarly reported that GAPDH-mediated interaction with human plasminogen system turns on GBS surface proteolytic activity and enhances bacterial virulence in a mouse model [21]. It remains to be demonstrated whether pilus proteins, the major GBS surface proteins, are key players for GAPDH interactions *in vivo*.

A mechanism explaining how these cytoplasmic proteins became surface associated was proposed by Bergmann *et al*. who demonstrated that the pneumococcal enolase protein reassociated on the bacterial surface after secretion [30]. The reassociation of secreted proteins without typical membrane anchor seems to be a general phenomenon and has been observed with a number of other such proteins [3]. In agreement with this model of secretion/reassociation, we demonstrated that purified rGAPDH is able to bind heat-inactivated GBS cells in a dose dependent manner (Fig. 3A). In addition, we showed that rGAPDH can bind to various GBS serotypes, to other Gram-positive bacteria such as *S. pyogenes*, *L. lactis*, *S. aureus*, and even to the phylogenetically remote Gram-negative bacterium *E. coli* indicating that rGAPDH can interact with very diverse cell surfaces (Fig. 3B).

### Uncovering a new role for GBS GAPDH

GBS was shown to induce apoptosis of microglia [43], murine macrophages [33], and human epithelial pulmonary A549 cells [44]. In murine macrophages, two distinct pathways leading to apoptosis were induced by GBS. Early apoptosis was shown to be caspase independent [33], being rather mediated by the Ca^2+^-dependent cysteine proteases, calpains [45]. The GBS β-hemolysin was shown to be important in this early stage apoptosis [33]. In contrast, late apoptosis was characterized as being β-hemolysin independent [46], and characterized by the release of cytochrome c and the activation of caspase 3 and 9 [47]. In a separate study, GBS-induced late stage apoptosis of macrophages was also found to be nitric oxide dependent [48]. Recently, eukaryotic GAPDH have been implicated as a regulator of cell death that participates in the apoptosis of several cell lines (reviewed in [34], [35]). It was also previously suggested that *S. pyogenes* GAPDH can cause DNA condensation, a penultimate stage of apoptosis upon direct contact with human pharyngeal cells [49]. Here, we demonstrate for the first time that a prokaryotic GAPDH is able to induce apoptosis of immortalized and primary murine macrophages in a dose-dependent manner (Fig. 6). By immunoprecipitating GAPDH from culture supernatants of GBS, *S. pyogenes* and *S. aureus* we partially but significantly inhibited the apoptosis induced by these culture supernatants. As others factors, such as β-hemolysin have also been shown to induce macrophage apoptosis [33], this may explain the incomplete neutralization of the apoptotic effect of GAPDH-depleted GBS supernatant. These results extend our findings on the role of GAPDH as a potent inducer of macrophage apoptosis to two other important human pathogens, i.e. *S. pyogenes* and *S. aureus*. Our results, by demonstrating that GBS GAPDH is another key element for GBS-mediated induction of apoptosis, ascribe to this enzyme a new role independent from its glycolytic function. Further studies aiming at characterizing the underlying mechanisms of apoptosis induced by GBS GAPDH will be carried out in the future.

GBS GAPDH is a surface-exposed moonlighting protein. We provide strong evidence that GAPDH is non-specifically released in the culture medium as a result of cell lysis and reassociates to the bacterial surface. Our results also suggest that penicillin G, a bacteriolytic antibiotic commonly used to treat GBS infections may have a Janus-faced behavior, by killing the pathogen and by increasing its virulence through enhanced GAPDH release. Furthermore, a new role for this protein beside its previous immunomodulatory activities has been uncovered in this study, i.e. inducer of murine macrophages apoptosis.

## Methods

### Ethics statement

All of the animal experiments described in the present study were conducted in accordance with guidelines of Cochin Institute, in compliance with the European animal welfare regulation (http://ec.europa.eu/environment/chemicals/lab_animals/home_en.html) and were approved by the Institut Cochin animal care and use committee.

### Bacterial strains and growth conditions

The main characteristics of bacterial strains and plasmids used in this study are listed in Table S1. *E. coli* and *S. aureus* were cultured in Trypticase soy (TS) broth or agar. GBS, *S. pyogenes and L. lactis* were cultured in Todd-Hewitt (TH) broth or Columbia agar supplemented with 5% horse blood (COH). Antibiotics were used at the following concentrations: for *E. coli*, kanamycin was used at 50 µg/mL and erythromycin at 150 µg/mL; for GBS kanamycin was used at 500 µg/mL and erythromycin at 10 µg/mL. All incubations were performed at 37°C.

### Mice

Wild-type C57BL6/J female mice, 8–12 weeks old, were purchased from Charles River Laboratories.

### General DNA techniques

Standard recombinant techniques were used for nucleic acid cloning and restriction analysis [50]. Plasmid DNA from *E. coli* was prepared by rapid alkaline lysis using the QIAprep Spin Miniprep Kit (Qiagen). Genomic DNA from *S. agalactiae* was prepared using the DNeasy Blood and Tissue Kit (Qiagen). PCR was carried out using *Pfu* turbo DNA polymerase (Agilent Technologies) or AmpliTaqGold DNA polymerase (Applied Biosystems) according to the manufacturer's specifications. Amplification products were purified with QIAquick PCR Purification Kit (Qiagen) and verified by sequencing.

### Construction of the deletion mutant *PilA/C^−^*

This double mutant was constructed as previously described [23] by replacing *pilC* into the NEM316:PilA^−^ *with a pilC allele* interrupted with a kanamycin-resistance gene.

### Overexpression constructs

The promoter region and the *gbs0093* coding region (http://genolist.pasteur.fr/SagaList/) were amplified with the primer pairs gbs0093_BamHI (GTTATggatccTGGCATTACCGCTATGTTGG) and gbs0093_PstI (AAACCctgcagCCTCTCGGTCTTTCTGAAAT) (the restriction sites used for cloning are written in lower case). The resulting amplicon was cloned in pCR-Blunt (Invitrogen) to originate pCR-BluntΩ*gbs0093.* This latter was digested with *Spe*I and *Xho*I, and ligated into the inducible vector pMSP3545 to give pMSP3545Ω*gbs0093*. This plasmid and pMSP3545 were electroporated in GBS NEM316 WT or PilB^−^ to generate strains overexpressing *gbs0093*. These strains were cultured until OD_600 nm_ = 0.3 and *gbs0093* expression was induced overnight with 20 ng/mL of nisin (Sigma-Aldrich).

### Growth curves

Overnight cultures of GBS strain NEM316 (WT), PilB^−^ and PilA/C^−^ in TH broth were diluted in fresh media at different concentrations: 1/50 to 1/500 and 150 µl were distributed in 96 wells plate with 5 replicates per strain tested. The microplate was incubated at 37°C with constant shaking in the BioTek Synergy plate reader and the OD_600 nm_ was recorded every 20 min for 7 h.

### Immunogold electron microscopy

For scanning electron microscopy (SEM) analysis, bacteria were fixed and stained with rabbit anti-rGAPDH IgG pAb followed by anti-rabbit secondary antibody conjugated to 10 nm colloidal gold as previously described [23].

### Supernatant and total protein extracts

Bacteria were grown in TH medium at 37°C and harvested for protein analysis during exponential (OD_600_ = 0.4) or stationary (OD_600_ = 0.9) phase of culture. For the preparation of culture supernatants used in the apoptosis experiments, bacteria were grown (OD_600_ = 0.9) in RPMI without phenol red supplemented with 0.1 M HEPES, 1% Glucose, amino acids solution (1×) and vitamins solution (1×) (Sigma-Aldrich). The culture supernatant was collected, filtered through 0.2 µm, and concentrated 50 times using Amicon® Ultracel 10 k centrifugal filters (Millipore) in the presence of a complete protease inhibitor cocktail (Roche Diagnostics). Total protein extracts from bacteria were prepared as previously described [23].

### Western Immunoblots

For analysis of GAPDH expression, bacterial proteins were separated into supernatant and total protein extracts (see above). Proteins were resolved on SDS-PAGE gels and transferred to nitrocellulose membrane (Pall). Detection of GAPDH [20], Nox-2 [27], SodA, DltA, and CAMP were performed using rabbit-specific polyclonal antibodies available in the laboratory. Horseradish peroxidase (HRP)-coupled goat anti-rabbit secondary antibody (Zymed) was added and detection was performed with enhanced chemiluminescence (ECL Reagent, GE Healthcare).

### Fluorescence-activated cell sorter analysis (FACS)

To analyze surface-exposed GAPDH, bacteria were collected and washed twice in phosphate buffered saline (PBS) before fixation in PBS containing 1% paraformaldehyde. After the incubation period of 20 min at 4°C, fixed bacteria were then washed twice with PBS and incubated for 45 min with rabbit anti-rGAPDH IgG diluted in PBS with 1% BSA or normal rabbit IgG at room temperature. After three washings with PBS, samples were incubated for 30 min with AlexaFluor488-conjugated goat anti-rabbit immunoglobulin (Molecular Probes, Invitrogen). Cells were washed and resuspended in PBS. Samples were acquired on a Beckman Coulter Cytomics FC500 apparatus and data were analyzed using Cytomics RXP software.

### ELISA binding assays

To analyze the interaction of rGAPDH with pili subunits, polystyrene microtiter plates (Nunc) were coated overnight at 4°C with 10 µg/ml of PilA, PilB, PilC (production of these recombinant proteins described in [23]) and rGAPDH or the domain S10 of HvgA [31] as controls. The wells were then saturated for 1 h with 2% BSA in PBS at room temperature. rGAPDH was added at 0, 6.25, 12.5, 25, 50, or 100 µg/mL and the plates were incubated for 2 h at room temperature. After washing 5 times in PBS containing 0.1% Tween 20 (Sigma-Aldrich), anti-rGAPDH IgG was added, incubated 2 h at room temperature followed by HRP-conjugated goat anti-rabbit antibody. After incubation for 1 h and extensive washing, the plates were revealed with a citrate solution containing o-Phenylenediamine dihydrochloride (OPD) (Sigma-Aldrich) and H_2_O_2_. After 20 min at room temperature, the reaction was stopped with 10% SDS and the absorbance was measured at 450 nm with a Biotek Chromoscan.

Interaction of rGAPDH with whole-GBS cells was analyzed as follows: 6.5×10^7^ CFU/mL of heat-inactivated NEM316 cells were seeded onto microtiter plates and incubated overnight at 4°C. After blocking 1 h with 2% BSA in PBS at room temperature, rGAPDH was added at various concentrations: 0, 10 or 50 µg/mL or rGAPDH at 50 µg/mL plus 75 µg of anti-rGAPDH IgG antibodies for 2 h at room temperature. After washing, mouse anti-pentaHis antibody (Qiagen) was added and the plates were revealed as described above.

### Live/Dead assay

To analyze the proportion of dead/live cells in a culture of NEM316 WT, PilB^−^ and PilA/C^−^ these strains were stained using LIVE/DEAD® *Bac*Light™. Bacterial viability and counting kit for flow cytometry (Molecular Probes, Invitrogen) according to the manufacturer's instructions. Briefly, 1 mL of bacterial suspension was washed in NaCl 0.9% and 10 µL of this were added to a FACS tube containing 1 µL of Syto9, 1 µL of propidium iodide (PI) and 978 µL of NaCl 0.9%. The same procedure was used to the 70% isopropanol treated cells that were used as control for dead cells. After incubation (15 min) in the dark at room temperature, 10 µL of beads were added to the tubes. Samples were acquired by a Beckman Coulter Cytomics FC500 apparatus and data were analyzed using Cytomics RXP software.

### Turbidity assays

GBS NEM316 and *S. aureus* were grown to exponential phase and the bacterial pellet obtained by centrifugation was washed and resuspended in PBS. The OD_600_ of both bacterial suspensions was recorded and arbitrarily set as 100%. These suspensions were incubated at 37°C without agitation and the decrease in OD was measured hourly for 5 h.

### Induction of lysis with Triton X-100 and Penicillin G

To analyze the GAPDH increase in the supernatant and surface of GBS after lysis induction, NEM316 WT or SodA^−^ were grown until exponential phase and 0.1% Triton X-100 and 15 U/mL of mutanolysin or PBS (control) were added. After incubation on ice for 45 min, the bacterial culture was centrifuged at 4000 *g*, the supernatant was collected and processed for immunoblotting analysis as mentioned above. The cell pellet was recovered, washed and stained for FACS analysis as detailed above. For treatment with Penicillin G, 5×10^6^ CFU/mL of NEM316 were treated for 12 h with Penicillin G at 100× MIC (6.4 µg/mL) or left untreated. After this time, the supernatant and cell pellet were collected for immunoblot and FACS analysis, respectively.

### rGAPDH reassociation assay

For analysis of the reassociation of rGAPDH to bacterial cells, 200 µg of rGAPDH or PBS (negative control) diluted in 500 µL of TH was added to 500 µL of an exponentially grown culture of several GBS strains belonging to different serotypes, *S. pyogenes*, *L. lactis*, *S. aureus* and *E. coli*. After an incubation period of 45 min at 37°C, total proteins were extracted by Fastprep and subjected to immunoblotting analysis.

### Cell culture techniques and apoptosis quantification by TUNEL assay

Murine macrophage cell line RAW264.7 was obtained from American Type Culture Collection and was used in passages between 13 to 16 for the assays. Bone marrow-derived macrophages from C57BL/6 mice were obtained and cultured as previously described [25]. RAW264.7 macrophages were seeded at 5×10^5^ cells/well in 24w cell culture Nunc plates 12 h before the assay. These macrophages were then treated for 24 h with 1 µM of staurosporin, 50 µg/mL of rGAPDH, or 200 µl of 50× concentrated supernatant from NEM316. We also used supernatant from other Gram-positive bacteria grown in the same conditions, i.e. *S. pyogenes*, *L. lactis* and *S. aureus* (strains are listed in Table S1). Supernatants were depleted from either GAPDH or SodA using the corresponding polyclonal antibody followed by immunoprecipitation with protein A Sepharose (GE Healthcare) according to manufacturer's instructions. As an additional immunoprecipitation control, culture supernatant was subjected to treatment with an irrelevant antibody (control IgG, normal rabbit IgG, Santa Cruz Biotechnology). Similarly, 5×10^5^ bone marrow-derived macrophages/well in 24w cell culture Nunc plates were treated for 24 h with 1 µM of staurosporin, various concentrations of GAPDH (5, 25, 50 µg/mL) or left untreated as negative control. In all treatments with rGAPDH or culture supernatant, polymyxin B was also added at 10 µg/mL to avoid side effects due to LPS contamination. After the treatments, both types of macrophages were detached gently using a cell scraper and stained using the DeadEnd Fluorometric TUNEL System (Promega) following the manufacturer's instructions. Samples were analyzed by FACS using a Beckman Coulter Cytomics FC500. For immunofluorescence staining, 5×10^5^ bone marrow-derived macrophages were seeded into 8-well culture slides (BD) and subjected to 50 µg/mL rGAPDH treatment or left untreated for 24 h as mentioned above. Following *in situ* TUNEL staining, the slides were visualized in a Zeiss Axiovert 200 microscope.

### Statistical analysis

Unpaired two-tailed t-test was used to analyze the differences between groups. A P value<0.05 was considered statistically significant.

## Supporting Information

Figure S1 Detection of GBS cytoplasmic proteins in NEM316 culture supernatant. Western Blotting of DltA, Nox-2, EF-Tu, SodA and GAPDH in total protein extracts (TP) or culture supernatants (SN) of NEM316 WT strain. 5 µg of NEM316 total proteins or 15 µL of each concentrated supernatant were loaded in the gel. After transfer to a membrane, proteins were detected using a polyclonal rabbit anti-anti-DltA, anti-Nox-2, anti-EF-Tu, anti-SodA or anti-rGAPDH IgG antibodies followed by HRP-conjugated goat anti-rabbit antibody.
(TIF)

Figure S2 Bacterial growth curves of GBS strains used in this study. Overnight cultures were diluted in fresh TH medium to give approximately 10^6^ CFU/ml. The inoculated broths were distributed (150 µl) in 96 wells plate incubated at 37°C with constant shaking in a plate reader and the OD_600_ was recorded every 20 minutes for 7 hours. Blank values (medium alone) were subtracted from experimental values to eliminate background readings. The y- axis is drawn in log10 scale. Results represent the mean and SD of 5 replicates.
(TIF)

Figure S3 The nisin inducible expression system in GBS NEM316. A) Effect of nisin on bacterial growth: pre-warmed TH broth containing or not nisin at the indicated concentration ranging from 31.25 to 500 ng/mL was inoculated with overnight NEM316/pMSP3545 strain to give approximately 10^7^ CFU/ml. The inoculated broth was distributed (150 µL) in 96 wells plate, incubated at 37°C with constant shaking in a plate reader and the OD_600_ was recorded every 20 minutes for 12 hours. Blank values (TH) were subtracted from experimental values to eliminate background readings. B) Production of the secreted staphylococcal nuclease NucB reporter induced by nisin: three nisin concentrations that did not affect bacterial growth were used to induce expression of NucB. Supernatant of overnight growth cultures of NEM316 containing pMSP3545 (control) or pMSP3545Ω*nucB* were collected by centrifugation and 10-fold concentrated by TCA precipitation. Equivalent of 100 µl of culture medium was spotted onto nitrocellulose membrane and analyzed for NucB content by dot-blot analysis using specific primary rabbit antibody.
(TIF)

Table S1 Bacterial strains and plasmids used in this study.
(DOC)
