# Supplementary figures and images for: Group B Streptococcus GAPDH Is Released upon Cell Lysis, Associates with Bacterial Surface, and Induces Apoptosis in Murine Macrophages
Source: PLoS One. 2012 Jan 23;7(1):e29963. doi: 10.1371/journal.pone.0029963 (PMC3264557; doi:10.1371/journal.pone.0029963)

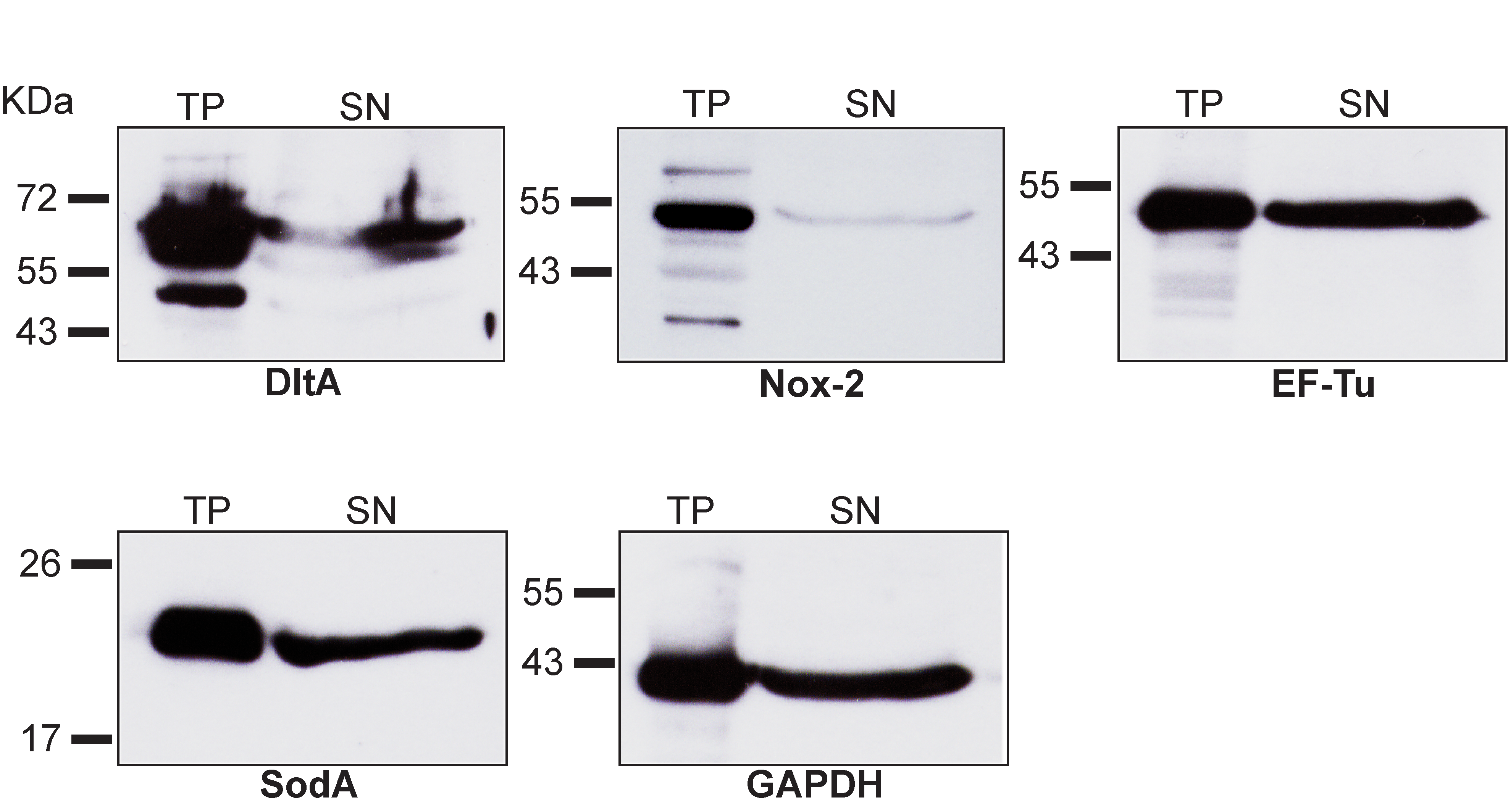

Supplement: Figure S1 — Detection of GBS cytoplasmic proteins in NEM316 culture supernatant. Western Blotting of DltA, Nox-2, EF-Tu, SodA and GAPDH in total protein extracts (TP) or culture supernatants (SN) of NEM316 WT strain. 5 µg of NEM316 total proteins or 15 µL of each concentrated supernatant were loaded in the gel. After transfer to a membrane, proteins were detected using a polyclonal rabbit anti-anti-DltA, anti-Nox-2, anti-EF-Tu, anti-SodA or anti-rGAPDH IgG antibodies followed by HRP-conjugated goat anti-rabbit antibody. (TIF) [file pone.0029963.s001.tif]

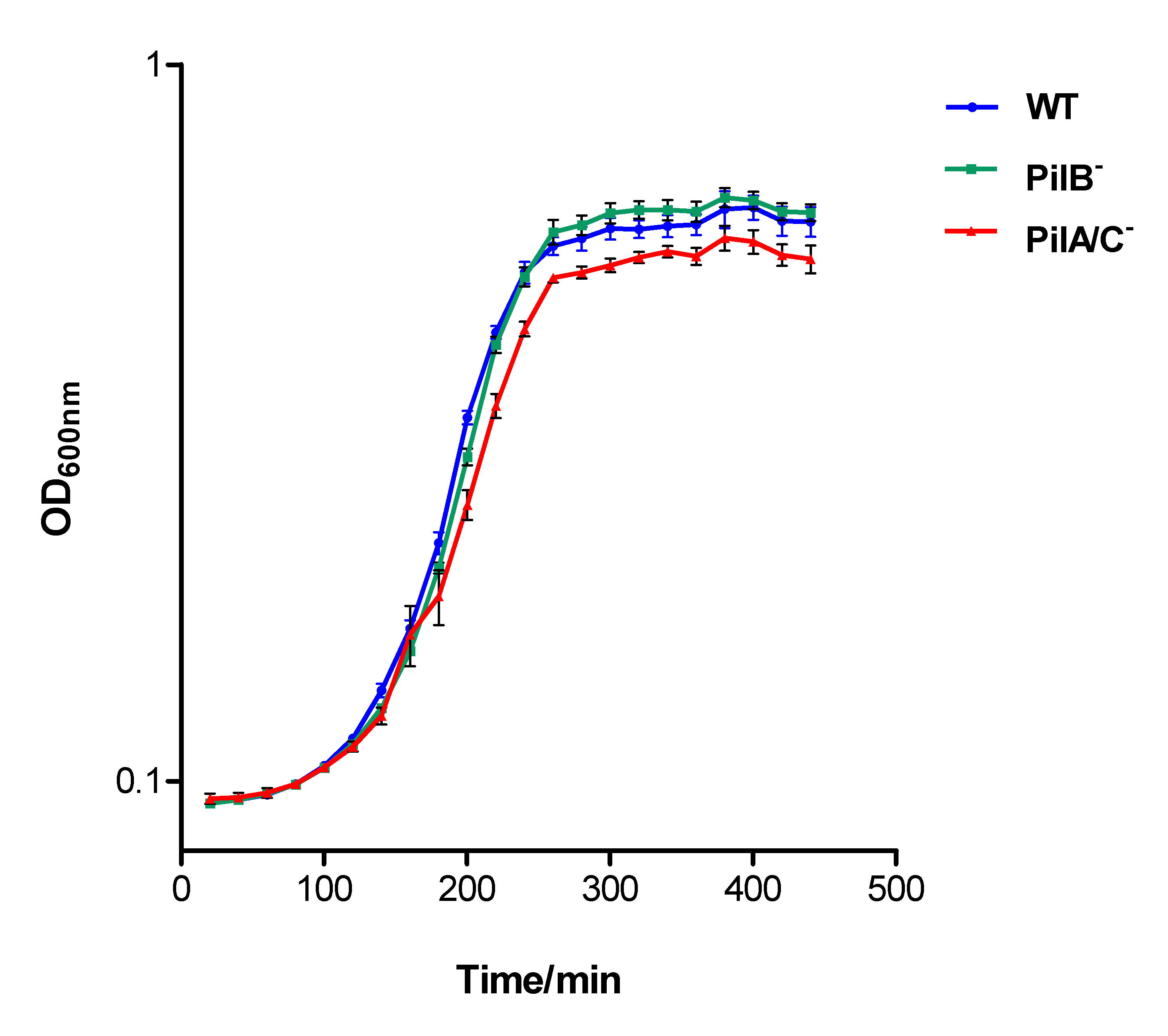

Supplement: Figure S2 — Bacterial growth curves of GBS strains used in this study. Overnight cultures were diluted in fresh TH medium to give approximately 106 CFU/ml. The inoculated broths were distributed (150 µl) in 96 wells plate incubated at 37°C with constant shaking in a plate reader and the OD600 was recorded every 20 minutes for 7 hours. Blank values (medium alone) were subtracted from experimental values to eliminate background readings. The y- axis is drawn in log10 scale. Results represent the mean and SD of 5 replicates. (TIF) [file pone.0029963.s002.tif]

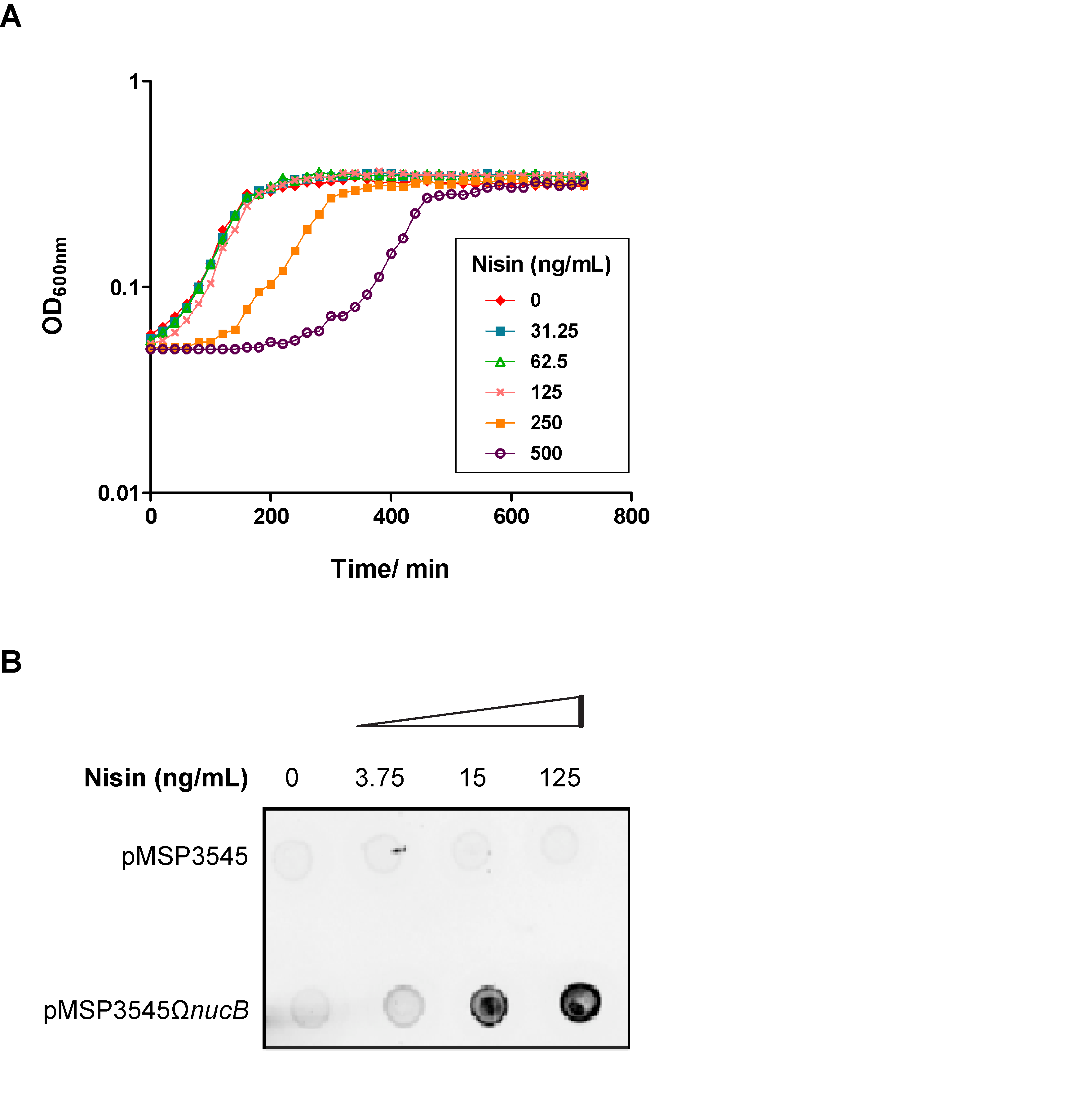

Supplement: Figure S3 — The nisin inducible expression system in GBS NEM316. A) Effect of nisin on bacterial growth: pre-warmed TH broth containing or not nisin at the indicated concentration ranging from 31.25 to 500 ng/mL was inoculated with overnight NEM316/pMSP3545 strain to give approximately 107 CFU/ml. The inoculated broth was distributed (150 µL) in 96 wells plate, incubated at 37°C with constant shaking in a plate reader and the OD600 was recorded every 20 minutes for 12 hours. Blank values (TH) were subtracted from experimental values to eliminate background readings. B) Production of the secreted staphylococcal nuclease NucB reporter induced by nisin: three nisin concentrations that did not affect bacterial growth were used to induce expression of NucB. Supernatant of overnight growth cultures of NEM316 containing pMSP3545 (control) or pMSP3545ΩnucB were collected by centrifugation and 10-fold concentrated by TCA precipitation. Equivalent of 100 µl of culture medium was spotted onto nitrocellulose membrane and analyzed for NucB content by dot-blot analysis using specific primary rabbit antibody. (TIF) [file pone.0029963.s003.tif]
