# Supplementary material for: Group B Streptococcus GAPDH Is Released upon Cell Lysis, Associates with Bacterial Surface, and Induces Apoptosis in Murine Macrophages
Source: PLoS One. 2012 Jan 23;7(1):e29963. doi: 10.1371/journal.pone.0029963 (PMC3264557; doi:10.1371/journal.pone.0029963)
Supplement: Table S1 — Bacterial strains and plasmids used in this study. (DOC) [file pone.0029963.s004.doc]

**Table S1. Bacterial strains and plasmids used in this study**

| Strains or Plasmid | Relevant properties | Source or reference |
| --- | --- | --- |
|  |  |  |
| **Strains** |  |  |
| *Escherichia coli* |  |  |
| DH5α | *recA1 gyrA* (Nal), Δ(*lacIZYA-argF*)[Φ80Δ*lac*Δ(*lacZ*)M15] | Invitrogen |
| DH1 | endA1 recA1 gyrA96 thi-1 glnV44 relA1 hsdR17(rK- mK+) λ- | [1] |
|  |  |  |
| *Streptococcus agalactiae* |  |  |
| A909 | Serotype Ia | [2] |
| H36B | Serotype Ib | [2] |
| 18RS21 | Serotype II | [2] |
| NEM316 | Serotype III ST-23 isolated from neonate blood culture | [3] |
| BM110 | Serotype III ST-17 isolated from neonate blood culture | [4,5] |
| CNR209-206 | Serotype IV | CNR^a^ |
| 2603V/R | Serotype V | [6] |
| CNR209-204 | Serotype VI | CNR^a^ |
| CNR209-207 | Serotype VII | CNR^a^ |
| NEM2398 | NEM316, PilB^-^ | [7] |
| NEM2655 | NEM316, PilA/C^-^ | This work |
| NEM1640 | NEM316, SodA^-^ | [8] |
| CCH1068 | NEM316, pMSP3545 | This work |
| CCH1070 | NEM316, pMSP3545Ω*gbs0093* | This work |
| CCH1072 | NEM2398, pMSP3545 | This work |
| CCH1074 | NEM2398, pMSP3545 Ω*gbs0093* | This work |
|  |  |  |
| *Streptococcus pyogenes* |  |  |
| SF370 | M1 serotype isolated from an infected wound | Kindly provided by E. Charpentier [9] |
|  |  |  |
| *Lactococcus lactis subsp. cremoris* |  |  |
| MG1363 | Lac - Prt - ; NCDO 712 derivative | [10] |
|  |  |  |
| *Staphylococcus aureus* |  |  |
| Newman | Isolated from a human infection | [11] |
|  |  |  |
| **Plasmids** |  |  |
| pG^+^host5 | Erm^R^, ColE1 replicon, thermosensitive derivative of pGK12; MCS pBluescript | [1,12] |
| pCR-Blunt | Kan^R^*, Sh ble;* pUC origin; *lacZα-ccdB;* T7 promoter | Invitrogen |
| pMSP3545 | Erm^R^, nisin-inducible gene expression (NICE-based plasmid); *E. coli*-Gram-positive shuttle plasmid | [13] |

1. Strains belonging to the collection of the Centre National de Référence des Streptocoques.

**REFERENCES**

1. Meselson M, Yuan R (1968) DNA restriction enzyme from *E. coli*. Nature 217: 1110-1114.

2. Lancefield RC, McCarty M, Everly WN (1975) Multiple mouse-protective antibodies directed against group B streptococci. Special reference to antibodies effective against protein antigens. J Exp Med 142: 165-179.

3. Glaser P, Rusniok C, Buchrieser C, Chevalier F, Frangeul L, et al. (2002) Genome sequence of *Streptococcus agalactiae*, a pathogen causing invasive neonatal disease. Mol Microbiol 45: 1499-1513.

4. Musser JM, Mattingly SJ, Quentin R, Goudeau A, Selander RK (1989) Identification of a high-virulence clone of type III *Streptococcus agalactiae* (group B Streptococcus) causing invasive neonatal disease. Proc Natl Acad Sci U S A 86: 4731-4735.

5. Stalhammar-Carlemalm M, Stenberg L, Lindahl G (1993) Protein rib: a novel group B streptococcal cell surface protein that confers protective immunity and is expressed by most strains causing invasive infections. J Exp Med 177: 1593-1603.

6. Tettelin H, Masignani V, Cieslewicz MJ, Eisen JA, Peterson S, et al. (2002) Complete genome sequence and comparative genomic analysis of an emerging human pathogen, serotype V *Streptococcus agalactiae*. Proc Natl Acad Sci U S A 99: 12391-12396.

7. Dramsi S, Caliot E, Bonne I, Guadagnini S, Prevost MC, et al. (2006) Assembly and role of pili in group B streptococci. Mol Microbiol 60: 1401-1413.

8. Poyart C, Pellegrini E, Gaillot O, Boumaila C, Baptista M, et al. (2001) Contribution of Mn-cofactored superoxide dismutase (SodA) to the virulence of *Streptococcus agalactiae*. Infect Immun 69: 5098-5106.

9. Gratz N, Siller M, Schaljo B, Pirzada ZA, Gattermeier I, et al. (2008) Group A streptococcus activates type I interferon production and MyD88-dependent signaling without involvement of TLR2, TLR4, and TLR9. J Biol Chem 283: 19879-19887.

10. Wegmann U, O'Connell-Motherway M, Zomer A, Buist G, Shearman C, et al. (2007) Complete genome sequence of the prototype lactic acid bacterium *Lactococcus lactis* *subsp. cremoris* MG1363. J Bacteriol 189: 3256-3270.

11. Baba T, Bae T, Schneewind O, Takeuchi F, Hiramatsu K (2008) Genome sequence of *Staphylococcus aureus* strain Newman and comparative analysis of staphylococcal genomes: polymorphism and evolution of two major pathogenicity islands. J Bacteriol 190: 300-310.

12. Biswas I, Gruss A, Ehrlich SD, Maguin E (1993) High-efficiency gene inactivation and replacement system for gram-positive bacteria. J Bacteriol 175: 3628-3635.

13. Bryan EM, Bae T, Kleerebezem M, Dunny GM (2000) Improved vectors for nisin-controlled expression in gram-positive bacteria. Plasmid 44: 183-190.
